# Long-Term Stable Thermal Emission Modulator Based on Single-Walled Carbon Nanotubes

**DOI:** 10.1021/acsami.3c06952

**Published:** 2023-07-31

**Authors:** Dezhuang Ji, Xuan Li, Moh’d Rezeq, Wesley Cantwell, Lianxi Zheng

**Affiliations:** † Department of Mechanical Engineering, Khalifa University of Science and Technology, P.O. Box 127788, Abu Dhabi 127788, United Arab Emirates; ‡ Department of Physics, Khalifa University of Science and Technology, P.O. Box 127788, Abu Dhabi 127788, United Arab Emirates; § System on Chip Center, Khalifa University of Science and Technology, P.O. Box 127788, Abu Dhabi 127788, United Arab Emirates; ∥ Department of Aerospace Engineering and Aerospace Research and Innovation Center (ARIC), Khalifa University of Science and Technology, P.O. Box 127788, Abu Dhabi 127788, United Arab Emirates

**Keywords:** infrared modulation, thermal emission, ionic gating, thermal camouflage, infrared display

## Abstract

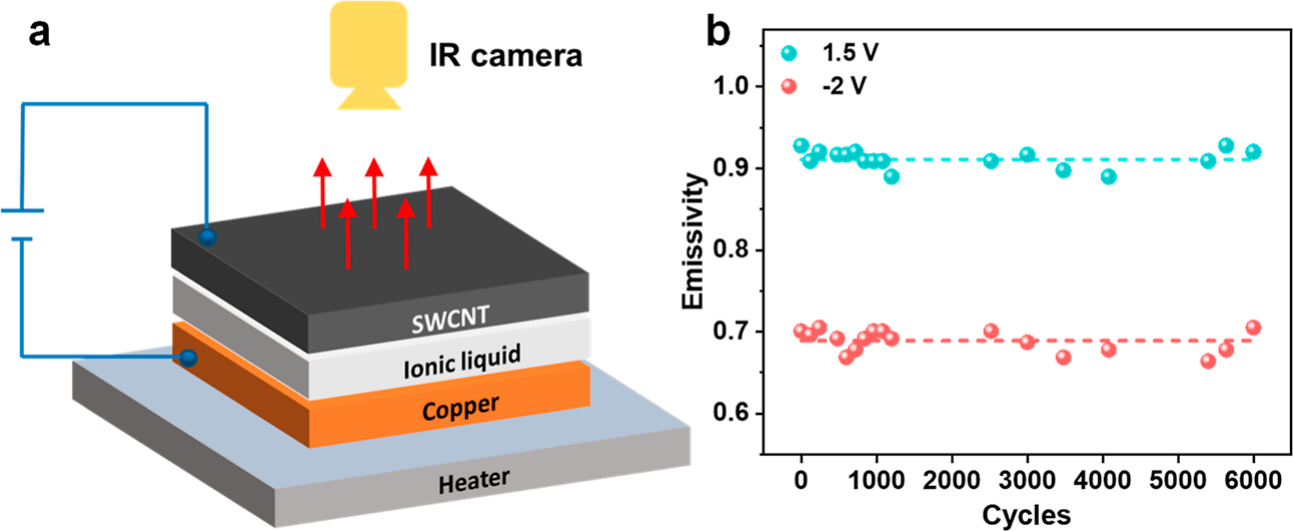

Dynamic control of a material’s thermal emission could enable many emerging applications, such as thermal camouflage and infrared (IR) display. Low-dimensional carbon nanomaterials have shown great potential in these applications because of their tuneability in charge density via static gating or ionic intercalation. Herein, a thermal emission modulator based on single-walled carbon nanotubes (SWCNTs) is realized by ionic gating. The Fermi energy of the SWCNTs is shifted via the adsorption of ions on the surface, and the highest emissivity is observed at the neutral state while both P-type and N-type SWCNTs have a reduced emissivity. An emissivity modulation range is achieved approximately from 0.45 to 0.95 within the electrochemical window of the used ionic liquid. Thermal camouflage and IR display applications are then demonstrated by utilizing the tuneable thermal emissivity of the fabricated SWNCT films. More importantly, a single-layer structure allows effective dynamic control purely by static gating, without involving any ion interaction process that may cause structural damage, as observed in graphene and multi-walled nanotubes. Therefore, the SWCNT-based IR modulators exhibit long-term stability, with nearly identical modulation range and response time after 6000 dynamic tuning cycles, indicating great potential for practical applications.

## 1 Introduction

It is of great scientific and practical interest to modulate a material’s optical properties, especially thermal emission in infrared (IR) regions, because of the emerging application demands in thermal camouflage^1,2^ and radiative cooling.^3−6^ The thermal emission of a surface is governed by the Stefan–Boltzmann law *P* = εσ*T*^4^, which is mainly dependent on the surface temperature, with the emissivity ε as the only material parameter that could be tailored. The conventional technique in altering the thermal emission is to use phase change materials^7−10^ or photonic crystals^11−13^ to selectively change the emissivity of the structures, but this technique could not realize the dynamic control due to their passive design and slow response in structural changes.

On the other hand, electrochromism is a phenomenon where the color or other optical properties of a material is reversibly changed under a small electric potential,^14^ which is a very effective route for dynamic control. It has wide applications in electrochromic display,^15^ smart windows,^16−18^ and sensing architectures.^14^ Carbon nanomaterials have shown promising properties in these applications due to their special electronic structures and ease in tuning their Fermi energy. Their optical property could be effectively tuned through filling or depleting the electrons from its Van Hove singularities. A number of studies have shown that the optical absorption could be changed dynamically by electrochemical doping,^19−21^ and the modulation speed as well as modulation depth could be efficiently enhanced through optimization of the electrode materials.^22,23^ Consequently, carbon nanomaterial-based electrochromism has been realized for applications in multiple spectral ranges, including color changes in visible range,^20^ optical imager,^24^ and modulator^25^ in terahertz ranges.

Therefore, it is very promising to obtain a thermal-emission modulator if such a dynamic control capability of carbon nanomaterials could be extended to IR regions. Previously, emissivity control via tuning the Fermi surface was studied in graphene,^26,27^ and the mid-IR modulator was attempted by using multi-walled carbon nanotubes (MWCNTs).^28^ However, due to the multi-layered structures of used materials, the ionic gating effect was only efficient in a few atomic layer thickness, so these methods involved very delicate growth processes and material-transfer processes to control the film thickness and achieve the effective gating effect. Another serious issue of using graphene and MWCNTs is that the gating effect involves the intercalation of ions into the inter-layers of materials, which induces irreversible structural damage and rapid decline in the performance. Significant deterioration in modulation range and response time has been observed in graphene and MWCNT-based modulators due to intercalation-related issues.^26−28^

Compared to MWCNTs and graphene, SWCNTs have much less possibility to involve ion interaction, which could eliminate irreversible structural damage and performance deterioration. A single-layer structure also allows easy and fast ion access/withdraw to/from the surfaces, making the gating effect much more effective and thus less requirements on thickness control. Therefore, in this work, we study the dynamic control of the SWCNT-based IR emission modulator. A facile vacuum filtration method is employed to prepare SWCNT films, and the filtering membrane is directly used as the porous media to contain ionic liquid (IL) in fabricating the mid-IR emission modulator. This approach could greatly ease the device fabrication process. The dynamic control of emissivity is successfully realized via an electrolyte static gating scheme without involving ion intercalation. In situ Raman spectroscopy is performed, and no structural changes are observed at all applied voltages. The long-term stability test shows that the IR modulator maintains high performance even after 6000 dynamic cycles, without apparent change in modulation range and response time. Finally, the applications of thermal camouflage and IR display are demonstrated to show its potential in long-term practical applications.

## 2 Experimental Methods

### 2.1 SWCNT Film Preparation

The SWCNT film was fabricated through a facile vacuum filtration method. The SWCNT powder (Timenano with purity >90%) was dispersed into deionized (DI) water at a concentration of 0.2 mg/mL with the assistance of sodium dodecyl sulfate. Stirring was performed to ensure that the powder was fully mixed in the solution. After that, bath sonication (3 h) was used to fully disperse SWCNTs into DI water. The SWCNT dispersion was then centrifuged at 10,000 rpm for 10 min, and the upper 50% supernatant solution was carefully decanted. The collected solution was carefully poured into a vacuum filtration apparatus with Celgard 3501 membrane (functionalized as hydrophilic) as a filter. During the filtration process, the film was rinsed with DI water to remove the surfactant. The resultant SWCNT film on the membrane was dried before the assembly step. No transfer of SWCNT films was needed because the Celgard 3501 was directly used as the spacer. The thickness of fabricated SWCNT films is around 2 μm.

### 2.2 Assembly of the Infrared Modulator

An IL, 1-ethyl-3-methylimidazolium bis(trifluoromethylsulfonyl)imide ([EMIM] [TFSI]), was carefully dropped on copper (Cu) foil. A SWCNT film-coated spacer (Celgard 3501 membrane) was then attached to the Cu foil to absorb the IL. Special care should be taken to make sure that no excess IL is dropped and no direct contact of IL to the surface of SWCNT film. Thereafter, a silver wire was connected to the Cu foil, and another Ag wire was attached to the SWCNT film surface with silver paste for applying electrical voltages.

### 2.3 Infrared Modulation Test

The assembled device was placed on a hot plate, and a double sticky tape was used to guarantee a perfect contact between the device and the hot plate. A power source (GW INSTEK GPS-3303) was employed to supply different voltages to the device, to characterize its voltage dependence. A thermal imager (FLUKE Ti480 Pro) was used to take the IR images at different voltages. In the long-term stability test, another power source (Siglent SDG2122X) was employed to produce a square electrical signal with the amplitude of 1.5 and −2 V and a period of 30 s. The thermal imager was used to record videos at specific cycles with a frame rate of 9 per second. The response time and emissivity of the IR modulator were measured and calculated at a time resolution of 110 ms.

### 2.4 Characterization

Scanning electron microscopy (SEM) is used to characterize the surface morphology of SWCNT films, and it is conducted on JEOL JSM-7610F. X-ray diffraction (XRD) and X-ray photoelectron spectroscopy (XPS) are employed to do structural analysis. XRD is performed on Bruker D2 Phaser with the Cu tube, and XPS is performed on Escalate Xi+. The thickness of the film is measured using a Mitutoyo micrometer with a resolution of 0.001 mm. Witec Alpha 300 RAS was used to measure the Raman shift of the SWCNT film. In order to perform in situ Raman shift characterization, the device is carefully placed on the stage with the help of a glass slide, and the power source was connected once the device was stabilized. The laser is initiated right after the sample was at focus with white light. After that, the voltage was applied at different values, and their corresponding Raman shift data were recorded. The Ossila four-point probe was used to measure the conductivity of SWCNT films. The height of the stage was carefully tuned until a stable conductivity data were present to make sure the probes uniformly contact the surface of SWCNT films.

## 3 Results and Discussion

### 3.1 Infrared Emissivity Modulation

**Figure 1:**
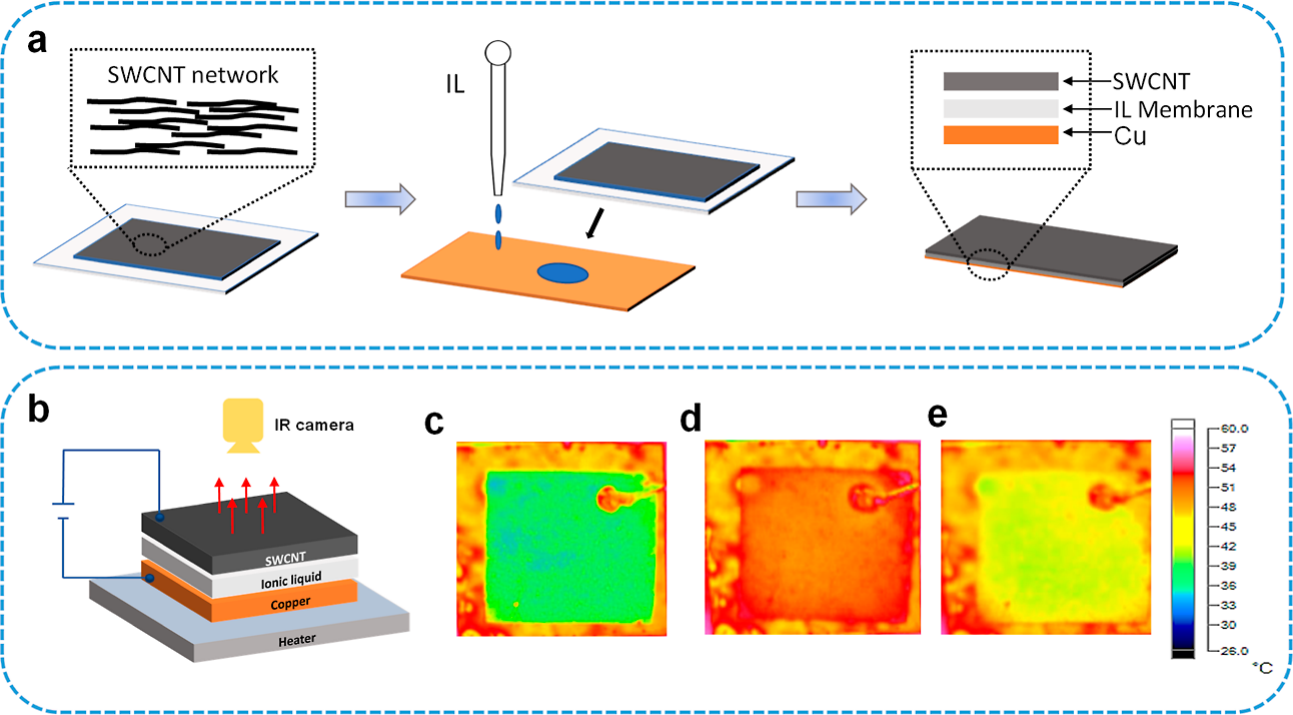
(a) Fabrication steps for an IR modulator. (b) Schematic structure of the IR modulator composed of SWCNT film and Cu electrode separated by the IL-filled spacer. (c) IR image of the SWCNT surface at −1.5 V, showing a surface temperature of 37 °C. (d) IR image of the SWCNT surface at +1 V, showing a surface temperature of 49 °C. (e) IR image of the SWCNT surface at +1.5 V, showing a surface temperature of 43 °C.

The fabrication steps of the device are schematically shown in Figure 1a. The SEM image of vacuum-filtrated SWCNT film exhibits a dense network of carbon nanotube bundles with diameter ranging from 12–63 nm (Figure S1a) and energy-dispersive spectrometry (EDS) mapping shows a high purity of carbon elements (Figure S1b,c). The emission modulator has a capacitor-like structure with the membrane filled with IL sandwiched between SWCNT film and Cu foil, structure of which is shown in Figure 1b. From the XRD and XPS analyses, the addition of IL does not lead to a structural and bonding change of SWCNT films (Figure S2). It is expected that the thermal emission of the SWCNT film could be modulated by external voltage via electrochemically ionic gating. The assembled device with the SWCNT film at upside was placed on a hot plate with a temperature around 55 °C, and then various voltages were applied (here and thereafter, the polarity of the voltage is in reference to the Cu gating electrode). The IR images of the SWCNT surface were recorded when the device reached the thermal equilibrium with the environment. As shown in Figure 1c–e, IR images from the SWCNT surface show different colors (related to the temperature scale in the IR camera) when different gating voltages are applied. The IR image of SWCNT surface shows a temperature of 37 °C when the voltage is set to −1.5 V (*i.e.*, Cu is negatively biased). The apparent temperature in the IR camera rises to 49 °C when the voltage is changed to +1 V. Thereafter, the reading temperature decreases to 43 °C when the voltage is increased to +1.5 V. In short, the IR camera reads different temperatures (apparent temperatures) from the surface of SWCNT films upon voltage bias, even the actual temperature of the SWCNT film is always the same during the whole process.

This is due to the fact that the IR camera measures the thermal radiation, which is the heat-transfer power from the surface of the object. According to Stefan–Boltzmann law, the radiated energy per unit area of a surface is *P* = εσ*T*^4^. If *T* is constant during this process, the parameter that has changed with the applied voltage is the intrinsic emissivity of the material. This change could be explained by electrochemical doping of the SWCNT film. When a voltage is applied, the cations and anions in IL will be driven oppositely, and one of them (depending on the voltage polarity) will accumulate on the surface of SWCNTs to build a strong electrical field, which induces the increase of the charge carriers with the opposite sign inside SWCNTs. This static charge accumulation effect, at the interface between IL and SWCNT, changes the Fermi level of SWCNTs, and thus change the IR absorption and emission. Therefore, a real-time control of the surface emissivity is realized by the electrical modulation. The detail mechanism and SWCNT band structure will be discussed in a later section.

**Figure 2:**
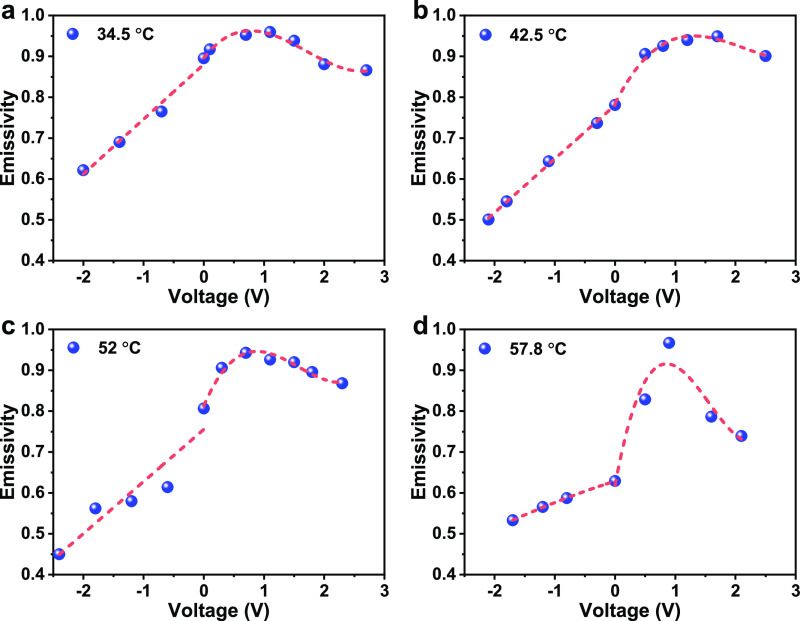
Voltage dependence of the emissivity at different real body temperatures of the SWCNT film (measured with thermocouple): (a) 34.5. (b) 42.5. (c) 52. (d) 57.8 °C.

Since the emissivity is the real parameter that changes according to the voltage, the apparent temperature recorded by the IR camera is then converted into the emissivity to study its detail dependence. It is known that IR camera measures the emitted thermal power and converts it to a temperature with a user set emissivity. According to the Stefan–Boltzmann law, the emissivity and temperature obey a power function to the emitted power. Therefore, the emitted power for a given image is fixed, and the actual emissivity value can then be obtained by matching the apparent temperature to the real temperature (measured by a thermocouple).^27^ The obtained emissivity is plotted against the applied gating voltage, as shown in Figure 2. The gating voltage is approximately tuned from −2 to 2.5 V considering the electrochemical window of [EMIM] [TFSI]^29^ and the breakdown voltage of the membrane.

As seen in Figure 2, the emissivity of the SWCNT film shows a similar overall tendency at various real body temperatures. It increases with the gating voltage, reaches the highest value (around 0.95) at a positive voltage of ∼1.1 V, and then decreases slightly at further increased voltages. The emissivity decreases continuously when a negative gating voltage is applied, and the lowest emissivity is seen at the highest negative voltage. The reason that the highest emissivity is observed at positive gating voltage of ∼1.1 V is because that the as-prepared SWCNT film is originally P-type, and a voltage is required to tune it back to the un-doped neutral state. The reason for asymmetric dependence of the emissivity is properly due to the different adsorption behaviors between cations and anions on SWCNT surfaces. Overall, the highest emissivity is observed at the neutral state of SWCNTs, while either P-type or N-type doping will lead to the decrease of emissivity, with the lowest emissivity observed from highly P-doped SWCNTs when a large negative gating bias is applied on the Cu electrode. The maximum modulation range is approximately from 0.45 to 0.95 (Figure 2c).

### 3.2 Tuning Range and Long-Term Stability

**Figure 3:**
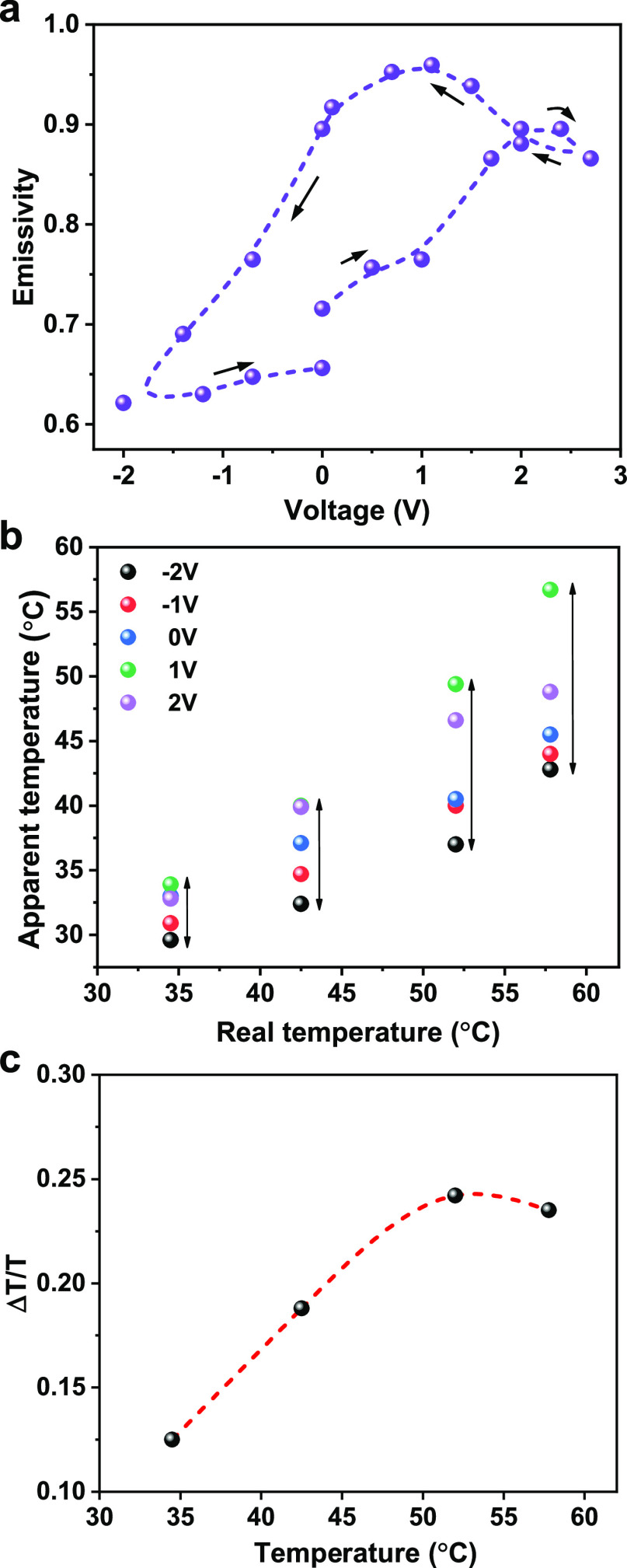
Cyclic and tuning range performance of the IR modulator. (a) Emissivity changes with cyclic voltage (sample at 34.5 °C). (b) Real temperature vs apparent temperature at voltages of −2, −1, 0, 1, and 2 V. (c) Relative tuning depth Δ*T*/*T* vs real temperature of the IR modulator.

To study the tuning ability and tuning range of the IR modulator, a complete voltage loop is applied to the device at 34.5 °C. The voltage is increased from 0 to 2.7 V, then decreased from 2.7 to −2 V, and finally increased from −2 to 0 V. The emissivity at each voltage is measured and is shown in Figure 3a. At the voltage range of 0–2.7 V, the emissivity increases first, reaches a small peak at around 2.2 V, and then decreases accordingly. As mentioned earlier, this is because of the fact that the SWCNTs are initially in P-type, which turn into neutral state and then become N-type. When the voltage is decreased from 2.7 to 0 V, the emissivity does not exactly follow the same path as the voltage was increasing, instead the neutral state (the highest emissivity) is seen at a voltage around 1.1 V. This difference could be explained by the viscous properties of ions in porous media: the movement of ions could not respond instantly to the external electrical field, which induced a hysteresis behavior, as seen in Figure 3a. For the same reason, the emissivity could not totally recover its original value at 0 V. When the voltage is further decreased from 0 to −2 V, the emissivity is decreased rapidly due to the increase of charge carriers resulted from the P-type doping effect. Finally, when the voltage is increased from −2 to 0 V, the emissivity increases gradually but does not return to its initial value because of the delayed ion movement. These hysteretic characteristics are also observed when the temperature of SWCNT is increased to 42.5 °C but disappear at higher temperatures. Nearly no hysteresis behavior is observed at 52 and 57.8 °C. This temperature dependence further confirms the role of IL’s viscosity in hysteresis behavior, *i.e.*, low temperature causes a relatively high viscosity that slows down the movement of ions under the electric field. Nevertheless, at the temperature above 50 °C, ions could respond to the electric field instantly, and the voltages for reaching the neutral state are almost identical (∼1.1 V).

The tuning ranges of the apparent temperature of SWCNT films are also studied against various real temperatures, with the voltage varying from −2 to 2 V. As seen in Figure 3b, the maximum apparent temperature is observed at ∼ 1 V while the lowest apparent temperature is obtained at −2 V. The range of tuneability is increased with the increase of the real temperature. A tuneable range of 14 °C is achieved at a real temperature of 57.8 °C, *i.e.*, for a device with the real temperature of 57.8 °C, one could tune its apparent temperature easily from 43 to 57 °C, which has great potential for the application of thermal camouflage. In addition, the relative tuning ability with respect to the body temperature is plotted in Figure 3c. It is observed that the relative tuning ability is increasing with increased temperature and peaks at around 52 °C, the value of which is 0.24. The relative tuning depth of our SWCNT-based modulator is reasonably large among all carbon nanomaterial-based modulators, as listed in Table 1.

**Table 1:** Emission Modulation Range and Stability Performance of Carbon Nanomaterial-Based IR Modulators

material	*T* (°C)	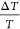	cycles	performance	year
graphene	55	0.2			2018^26^
graphene	70	0.25	780	reduction of 50% modulation depth	2019^27^
MWCNT	60	0.45	6000	reduction of 33% modulation depth	2021^28^
rGO	90	0.15			2021^31^
MWCNT	50	0.2	100	reduction of 23% modulation depth	2023^32^
SWCNT	100	0.09			2023^33^
This work	52	0.24	6000	nearly 0% reduction	

**Figure 4:**
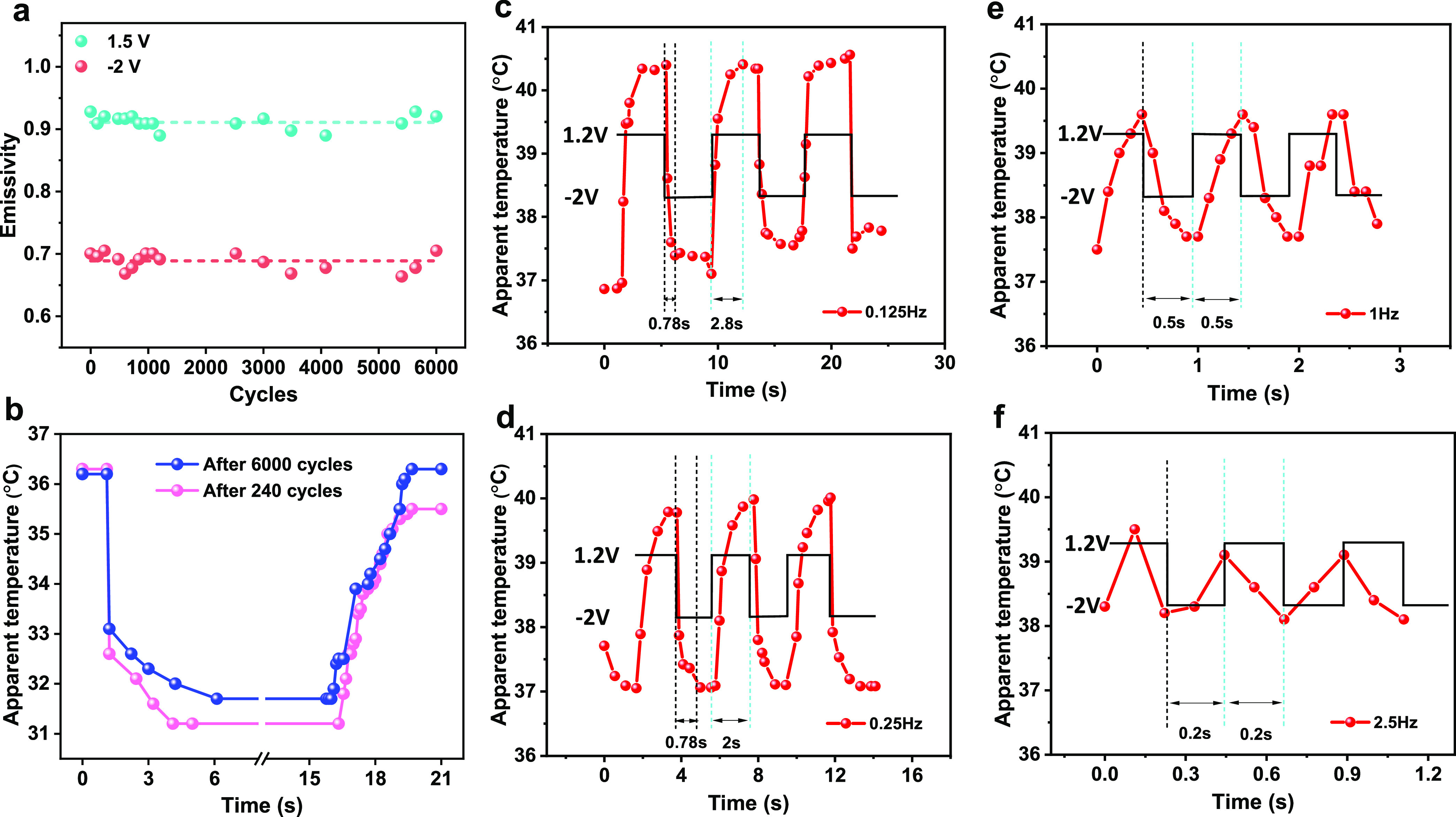
Long-term performance and response of IR modulator at different frequencies. (a) Long-term emissivity test at 39 °C (a square wave with a period of 30 s and magnitude of +1.5 and −2 V to tune the emissivity of SWCNTs), (b) transient response of the SWCNT film, recorded by a thermal camera of 110 ms each frame and with the same voltages used for the long-term test. The response of the IR modulator when a square signal with the amplitude of −2 V and 1.2 is applied. The frequencies of the square signal are (c) 0.125, (d) 0.25, (e) 1, and (f) 2.5 Hz.

Previous studies based on the intercalation of ions into different layers to change the Fermi level has a main concern on irreversible structural damage and performance deterioration. For examples, graphene-based modulators had a strong temperature-dependent but less than 750 cyclic lifetime;^27^ The MWCNT-based modulator showed almost 50% decrease in the tuning range and 15–24 times increase in the response times after 6500 cycles.^28^ The IR modulator made of SWCNTs is possibly more robust due to the elimination of ion intercalation. The cyclic performance is then exanimated with a square wave electrical signal, of which the period is 30 s and the amplitude is 1.5 to −2 V. As shown in Figure 4a, there is nearly no drop in the tuning range of emissivity even after 6000 cycles. The emissivity is nearly constant, with slight fluctuating around 0.91 for *V* = 1.5 V and 0.68 for *V* = −2.0 V, respectively. The transient response was also tested to verify possible degradation of the response profile, and the results are shown in Figure 4b. When the negative electrical signal is applied, the apparent temperature drops more than 60% of the tuning range within 110 ms and approaches to a steady state gradually. At the positive electrical cycle, the apparent temperature increases from one steady state to another state in around 4.5 s. This asymmetric response behavior is attributed to different dynamical properties of anions and cations, such as mobility or viscosity.^30^ More importantly, the transient response of apparent temperature is almost identical after 6000 cycles. The data of long-term performance of various IR modulator are also compared in Table 1, and our SWCNT IR modulator has the most stable long-term performance so far. The electrical conductivity of the SWCNT film was also measured before and after cyclic tests, with a value of 2.4 kS/m at 0 cycle and 2.5 kS/m after 6000 cycles, respectively, suggesting no structural damage. Overall, neither significant deviation in IR modulator’s performance nor irreversible structural damage after 6000 cycle is observed. This could be attributed to the unique structures of SWCNTs, which only involve ion adsorption without any intercalation-related issues observed in graphene or MWCNTs.

In addition, three samples of various thicknesses (less than 1, 1, and 12 μm) are prepared to study their effect on the tuning performance. A square wave signal with amplitude of 1.2 and −2 V is employed to modulate the apparent temperature of SWCNT films (Figure S3 and the corresponding values are recorded in Table S1). It is found that thickness has a significant influence on the tuning performance. First, the surface with a thin SWCNT film (less than 1 μm) has a high emissivity when no gating is applied. This is because that the IL-filled Celgard film has an intrinsic high emissivity, and the thin SWCNT film could not fully shield its IR emission. Second, the tuning range of the thin film is much smaller compared to 1 μm thick sample due to the fact that the ionic-filled Celgard film does not possess tuneability. On the other hand, the surface with a very thick SWCNT film (12 μm) has a lower emissivity, which approaches the intrinsic emissivity of SWCNTs. This thick film losses the tuneability completely because the ionic gating is only effective to a very thin region but 12 μm far exceeds the threshold. Therefore, a moderate thickness (1–2 μm) will give the largest emissivity tuneable range.

Response of our IR modulator at different tuning frequencies is also investigated. The IR modulator with 1 μm thick SWCNT film is placed on a hot plate of 42 °C and modulated with a square electrical signal (1.2 and −2 V). Four different tuning frequencies are applied, as shown in Figure 4c–f. It is observed that the apparent temperature could fully recover its initial state when the tuning frequency is low. The response time for tuning down the temperature is around 0.78 s while tuning up time is around 2.8 s. As the tuning frequency keeps increasing (the half period of the square tuning signal becomes shorter than the response time of the IR modulator), the apparent temperature could not be fully recovered, and the tuning range is decreasing accordingly. The tuning range vs frequency is summarized in Figure S4 (it is worth noting that the response time also depends on the temperature and the thickness of the SWCNT film). The real operation frequency could be selected according to the applications: low frequency to achieve maximum range in thermal camouflage while high frequency with reasonable contrast in displaying application.

### 3.3 In Situ Raman Spectroscopy Study

**Figure 5:**
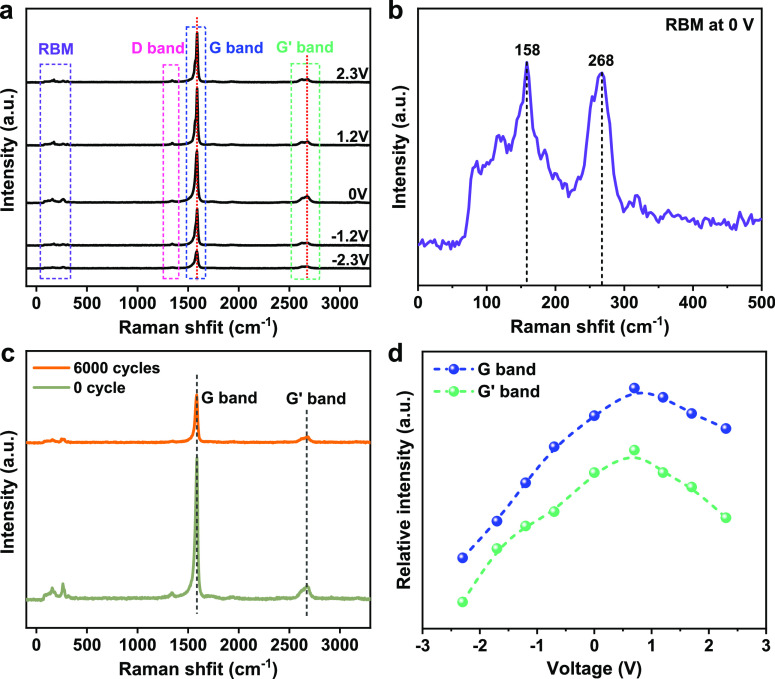
In situ Raman shift of SWCNTs. (a) Comparison of Raman shift of SWCNTs at −2.3, −1.2, 0, 1.2, and 2.3 V, (b) RBM peaks of SWCNTs at 0 V, (c) Raman shift of SWCNTs at 0 cycle and after 6000 cycles (0 V), and (d) voltage dependence of Raman intensities of G and G′ bands.

The in situ Raman shift was also utilized to check the structural integrity. Raman spectra were obtained with a laser of 532 nm for SWCNTs. As shown in Figure 5a, G bands is related to the sp^2^ bonding of carbons, G′ band is originated from a second-order process that involves two phonons, and D band is induced by disorder or defects. In a previous study for MWCNT-based IR modulators, a shift of G band was observed,^28^ which was attributed to the structural damage caused by ion intercalation. However, there is no obvious peak shift of G band and G′ band at different voltages for our samples (Figure 5a), which means no structural change for SWCNTs, and the ions only physically attach on the surface of carbon nanotubes during the tuning of Fermi energy. The existence of two radial breathing mode (RBM) peaks (158 and 268 cm^–1^) is the direct evidence that our CNT film is composed of SWCNTs with the radius mainly concentrated at ∼1.43 and ∼0.85 nm (Figure 5b). Together with the negligible D band, all evidence suggests very less defects in our SWCNTs and no defect was generated from applying voltages. In addition, the Raman shift of SWCNTs at 0 cycles and after 6000 cycles is compared at zero voltage (Figure 5c). As expected, no Raman shift of G and G′ band are observed, indicating long-term stable performance for IR modulation. It is also worth mentioning that the physical adsorption of ions on SWCNT surfaces could be monitored or evaluated by the intensities of G and G′ band. As shown in Figure 5d, both G band and G′ band change their intensities according to the applied voltages. This effect is induced by the phonon softening phenomenon^34^ because different voltages will lead to different doping states of SWCNTs. The highest intensity is observed at +0.7 V, which is close to the neutral state and also confirms that the SWCNTs are initially P-type. Both N-type and P-type SWCNTs have a lower Raman intensity in G and G′ bands.

### 3.4 Mechanism of Emission Modulation

**Figure 6:**
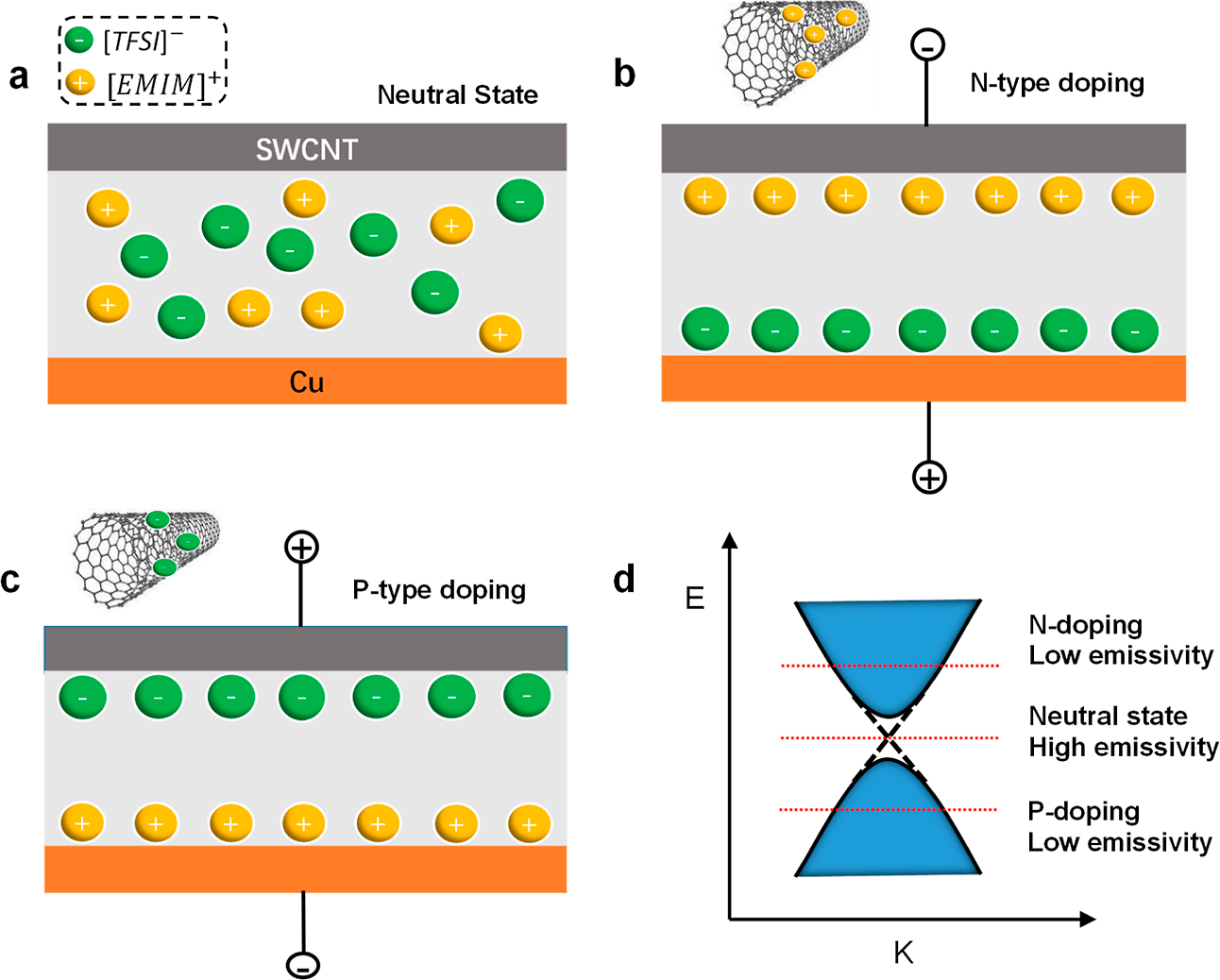
Schematics illustrate the mechanism of tuning the thermal emissivity. (a) State of ions when no voltage is applied, (b) state of ions when a positive gating voltage is applied to the Cu electrode, leading to the accumulation of positive ions on the surfaces of SWCNTs and inducing N-type doping in SWCNTs, (c) state of ions when a negative gating voltage is applied to the Cu electrode, leading to the accumulation of negative ions on the surfaces of SWCNTs and inducing P-type SWCNTs, and (d) illustration of Fermi energy levels and the emissivity of SWCNTs in each state.

The emissivity tuning process is realized through shifting the Fermi energy of SWCNTs. In general, there are two ways to shift the Fermi surface of a material: chemical doping and ionic gating. Chemical doping relies on the formation of chemical bonds to introduce excess electrons or holes; ionic gating (also named as electrochemical doping or electrochemical gating) is similar to the field effect and is tuning the charge densities through an external electric field. Compared to the chemical doping, ionic gating is reversible and thus could be used for real-time dynamic control. It could be realized via a capacitor-like structure, as illustrated in Figure 6. Specifically, the gating electrode is copper in our case. The positive ions will be driven to and accumulate on the surface of SWCNT when a positive gating voltage is applied. Since the gap between ions and SWCNT is at sub-nanometer, a very strong electric filed is exerted to SWCNT and then increases its density of electrons. Vice versa, negative gating voltage will increase the density of holes. The ionic gating process is schematically shown in Figure 6. The ions in the porous spacer membrane are randomly distributed in the initial state at zero external voltage (Figure 6a). However, once a positive gating voltage is applied, the cations will be driven to the SWCNT and accumulate on its surface, while anions accumulate on the surface of the gating electrode (Cu). Two thin capacitors will be formed at the interfaces between ions and electrodes, as shown in Figure 6b. Therefore, a very strong electric field is generated due to the extremely small spacing of the capacitor. This strong electric field will further increase the density of electrons within SWCNTs to achieve N-doping, *i.e.*, the Fermi level of SWCNTs will be shifted up at the positive gating voltage. Similarly, the Fermi level is shifted down when a negative gating voltage is applied (Figure 6c). As shown in Figure 6d, the cations will increase the Fermi level and lead to N-type doping, while anions will decrease the Fermi level and lead to the P-doping. Also according to the ref (19), emissivity could be evaluated using Kirchhoff’s law, *i.e.*, the emitted energy must be equal to the absorbed energy under equilibrium state. Therefore, shifting Fermi energy will suppress the interband transitions that contributes a major part to the energy absorption and thus decrease the emissivity. In this aspect, N-type and P-type states are the same in tuning principle: shifting Fermi energy away from its neutral state to suppress the interband transitions (in P-type state, the transition is suppressed because a higher energy is required for an electron to transit from deep valance band to the conduction band; while in the N-type, the transition is suppressed because the energy state in conduction band are occupied).^35,36^ Therefore, the emissivity is tuned according with the tuning of Fermi energy, with the largest emissivity at the neural state and reduced emissivity in both N-type and P-type states, due to the enhancement/suppression of interband transitions.

### 3.5 Emission Control for IR Display and Thermal Camouflage

**Figure 7:**
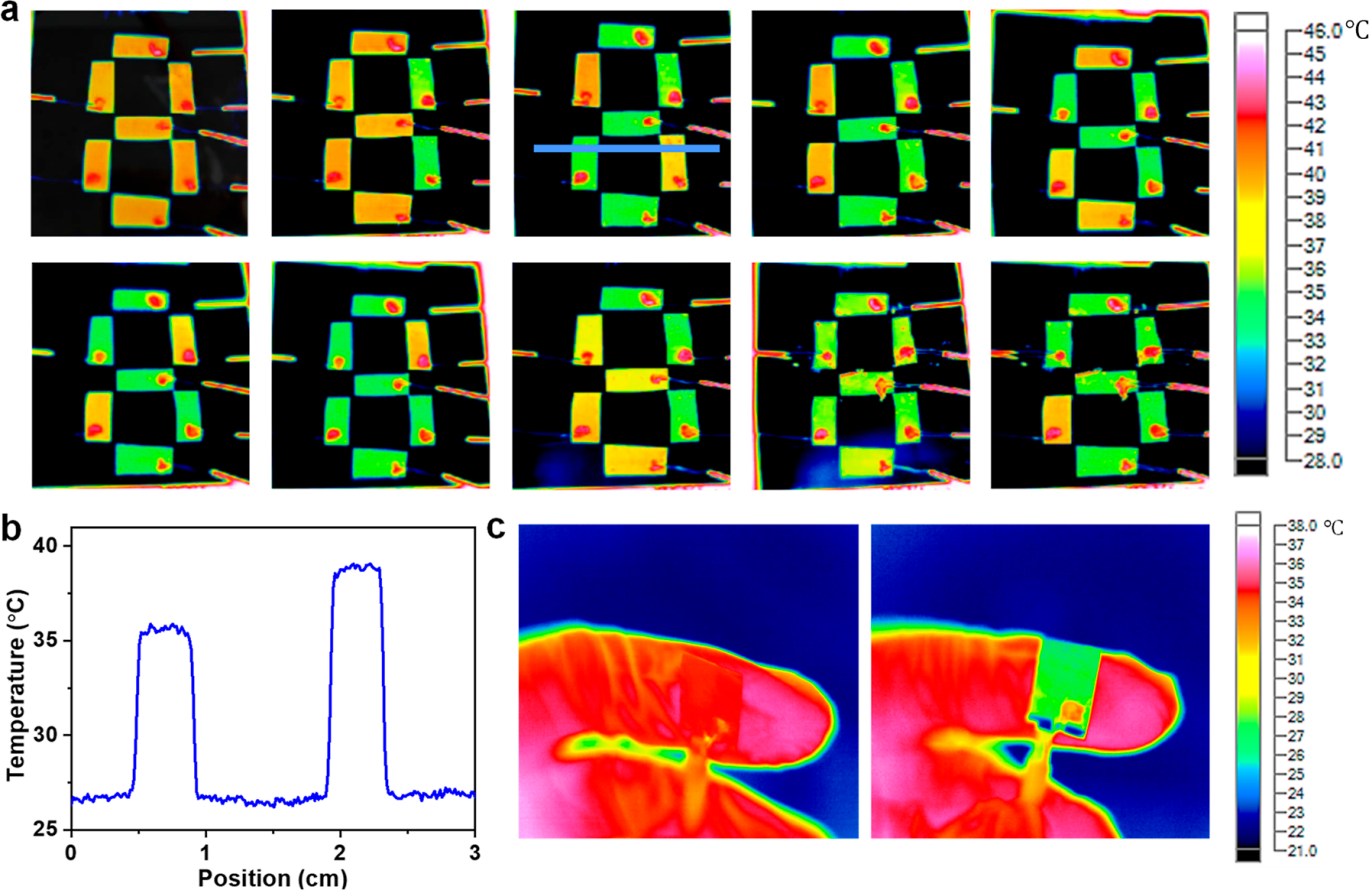
Applications of IR modulators. (a) Display of numbers, from 1 to 9, in the IR camera using −1.5 V to tune the emissivity of the stripes placed on a hot plate of around 45 °C, (b) temperature profile along the blue line shown in a, and (c) thermal camouflage effect on a human hand (left IR image with no bias and the right IR image with −2 V bias).

The capability of the fabricated devices in emission control could be used to display IR information. As shown in Figure 7a, the vacuum-filtered SWCNT film is cut into small stripes, and seven stripe-shaped modulators were carefully placed to show the number “8”. Both −1.5 and 0 V are used to tune the IR contrast that are corresponding to “ON” and “OFF” state of each stripe and collectively to show different numbers via various bias combinations. The background is nearly black which corresponds to a very low temperature according to the temperature scale because Cu has much lower emissivity (less than 0.1). Therefore, a real-time display of numbers in IR camera is shown (Figure 7a). The apparent temperature profile or image contrast of the digit “2” along the line shown in Figure 7a is exhibited in Figure 7b. It can be seen that Cu has a much lower temperature while the two SWCNT stripes with “ON” and “OFF” state have temperature difference, representing the contrast in the IR image.

In addition to the display of information, the fabricated device could also be used in a real-time thermal camouflage application, as shown in Figure 7c. The color in the IR image could be tuned with the applied voltage on the SWCNT film. The SWCNT film has a very high emissivity when no external voltage is applied (Figure 7c left), so it could be observed by the IR camera as a hot object (same as the hand). However, it yields a much-reduced emissivity when a −2 V external voltage is applied and shows a bluish color (much low apparent temperature) close to the environment. Therefore, a high temperature object (finger that covered by SWCNT films) could be hided in the IR camera upon applying an external voltage. In such applications of thermal camouflage, one should pay specific attention to the contact between the SWCNT film and the skin, because the variation in heat transfer will affect the real temperature of SWCNTs and thus shift the bias voltage.

## 4 Conclusions

An emission modulator based on SWCNTs is fabricated via a facile vacuum filtration method. The emissivity of SWCNTs could be easily controlled by a gating voltage in real time. The modulation range depends on the gating voltage and the actual temperature of the SWCNTs, with a maximum range from 0.45 to 0.95 being achieved within the electrochemical window of the electrolyte. The emissivity control is achieved via the suppression of interband transitions by tuning SWCNT Fermi energy, which is further realized through the absorption of ions on the surfaces of SWCNTs without involving any intercalation process. Consequently, the SWCNT-based modulator shows excellent long-term stability. Its modulation performance is nearly identical, with no decrease in modulation range and delay in the response time, after 6000 dynamic cycles. This is attributed to the absence of the ion intercalation process in the gating scheme and only the absorption of ions on the SWCNT surfaces, which significantly reduces the possibility of permanent structural damage that observed in graphene and MWCNT-based modulators. An in situ Raman study shows no peak shift of G and G′ band, confirming the structural integrity during the long-term operation. The fabricated IR modulators have also been demonstrated in the applications of information IR displaying and thermal camouflage.

## Supporting Information Available

XRD of SWCNT powder, Celgard 3501 film, SWCNT-coated Celgard 3501 film and IL-filled Celgard film coated with SWCNTs; XPS survey spectra and C1s spectra; dependence of tuning ability of the SWCNT emission modulator; apparent temperature of the modulator surfaces with different SWCNT film thicknesses and gating voltages; and dependence of tuning range with respect to the input tuning signal frequency (PDF)
