# Supplementary material for: Long-Term Stable Thermal Emission Modulator Based on Single-Walled Carbon Nanotubes
Source: ACS Appl Mater Interfaces. 2023 Jul 31;15(31):37818–27. doi: 10.1021/acsami.3c06952 (PMC10416147; doi:10.1021/acsami.3c06952)
Supplement: Supplementary file 1 — am3c06952_si_001.pdf [file am3c06952_si_001.pdf]

Supporting information

Long-term Stable Thermal Emission Modulator based on Single-walled Carbon Nanotubes

*Dezhuang Ji [a], Xuan Li [a], Moh'd Rezeq [b, c], Wesley Cantwell [d], Lianxi Zheng [a, *]*

[a] Department of Mechanical Engineering, Khalifa University of Science and Technology, P.O. Box 127788, Abu Dhabi, United Arab Emirates

[b] Department of Physics, Khalifa University of Science and Technology, P.O. Box 127788, Abu Dhabi, United Arab Emirates

[c] System on Chip Center, Khalifa University of Science and Technology, P.O. Box 127788, Abu Dhabi, United Arab Emirates

[d] Department of Aerospace Engineering and Aerospace Research and Innovation Center (ARIC), Khalifa University of Science and Technology, P.O. Box 127788, Abu Dhabi, United Arab Emirates

*Corresponding author: lianxi.zheng@ku.ac.ae (Lianxi Zheng)

Material characterization

The SEM image of the SWCNT film is shown in Figure S1a. It is seen that many SWCNT bundles (with diameter of 12-63 nm) intertwine together and form a network. Energy dispersive spectrometry (EDS) mapping of SWCNT film is also conducted (Figure S1b-c). The carbon account is 97.08% in weight, indicating high purity and less functional groups.

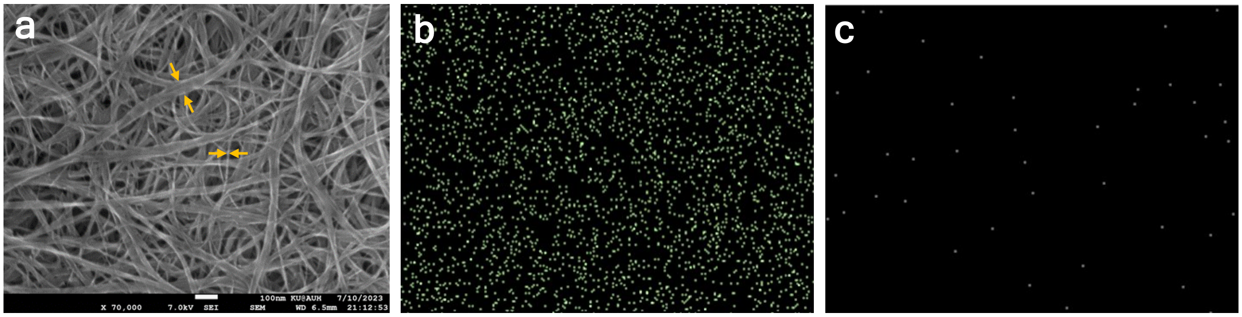

Figure S1. (a) Scanning electron microscopy (SEM) image of SWCNT film. Energy dispersive spectrometry (EDS) mapping of SWCNT film: (b) Carbon element, (c) Oxygen element.

The X-ray diffraction (XRD) characterization of the SWCNT powder shows a peak at $26°$ corresponding to (002) crystal plane, Celagrd 3501 membrane (surfactant coated porous polypropylene) exhibits characteristic peaks of 2θ at $14°$,$16.9°$, $18.5°$, and $25.4°$, corresponding to (110), (040), (130) and (060) crystal planes which is consistent with reference[1] (Figure S2a). However, the coating of SWCNT film on polypropylene does not exhibit a clear peak at 26°. The possible reason is probably due to the very thin thickness of SWCNT film (1-2 μm)[2].

In addition, X-ray photoelectron spectroscopy (XPS) is conducted for SWCNT film. It is observed that SWCNT exhibits characteristic peaks of C1s peak (284.5eV) and O1s (532.2eV), as shown in Figure S2b. The high content of C=C compared with other carbon bonding indicates a high graphitized crystallinity (Figure S2c). Moreover, analysis of O1s spectra implies the presence of oxygen containing functional groups (Figure S2d).

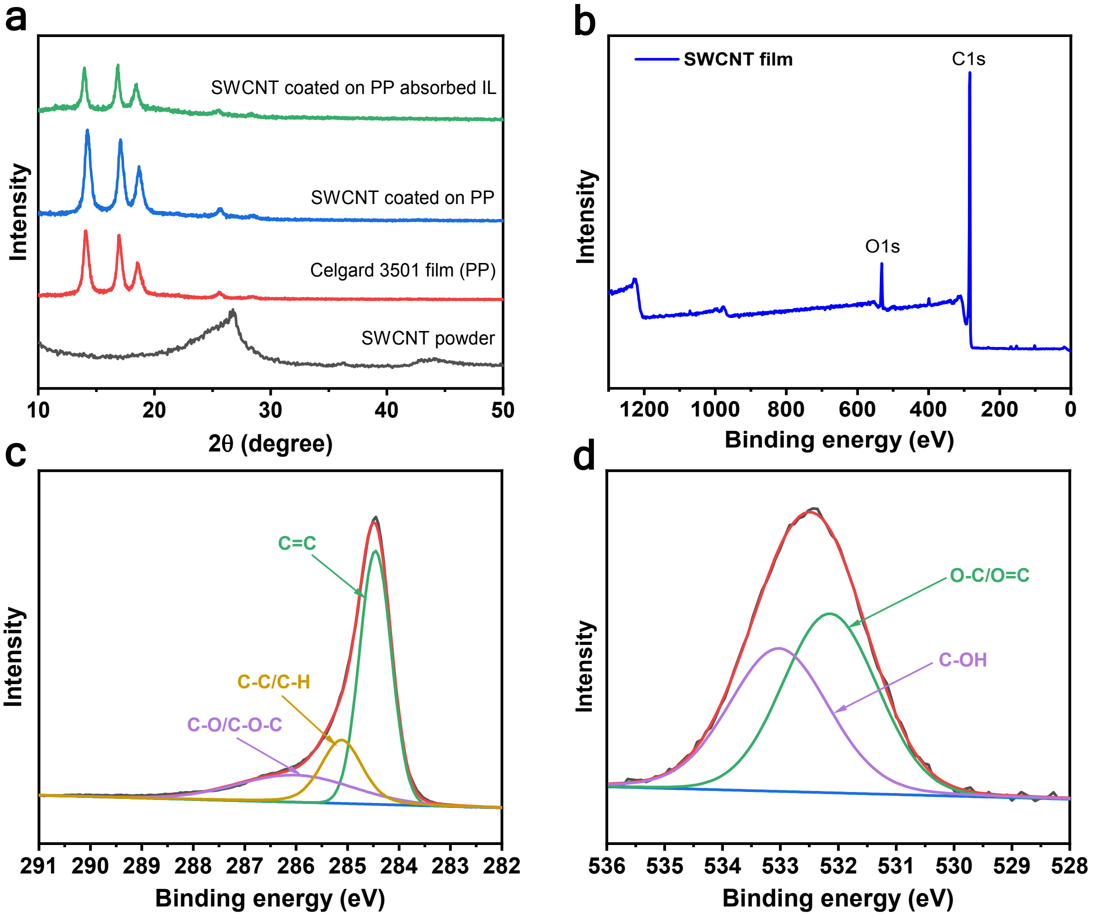

Figure S2. (a) XRD of SWCNT powder, Celgard 3501 film, SWCNT coated Celagrd 3501 film and IL filled Celgard film coated with SWCNT. (b) XPS survey spectra of SWCNT film. (c) C1s spectra of SWCNT. (d) O1s spectra of SWCNT.

Performance dependence on SWCNT film thickness

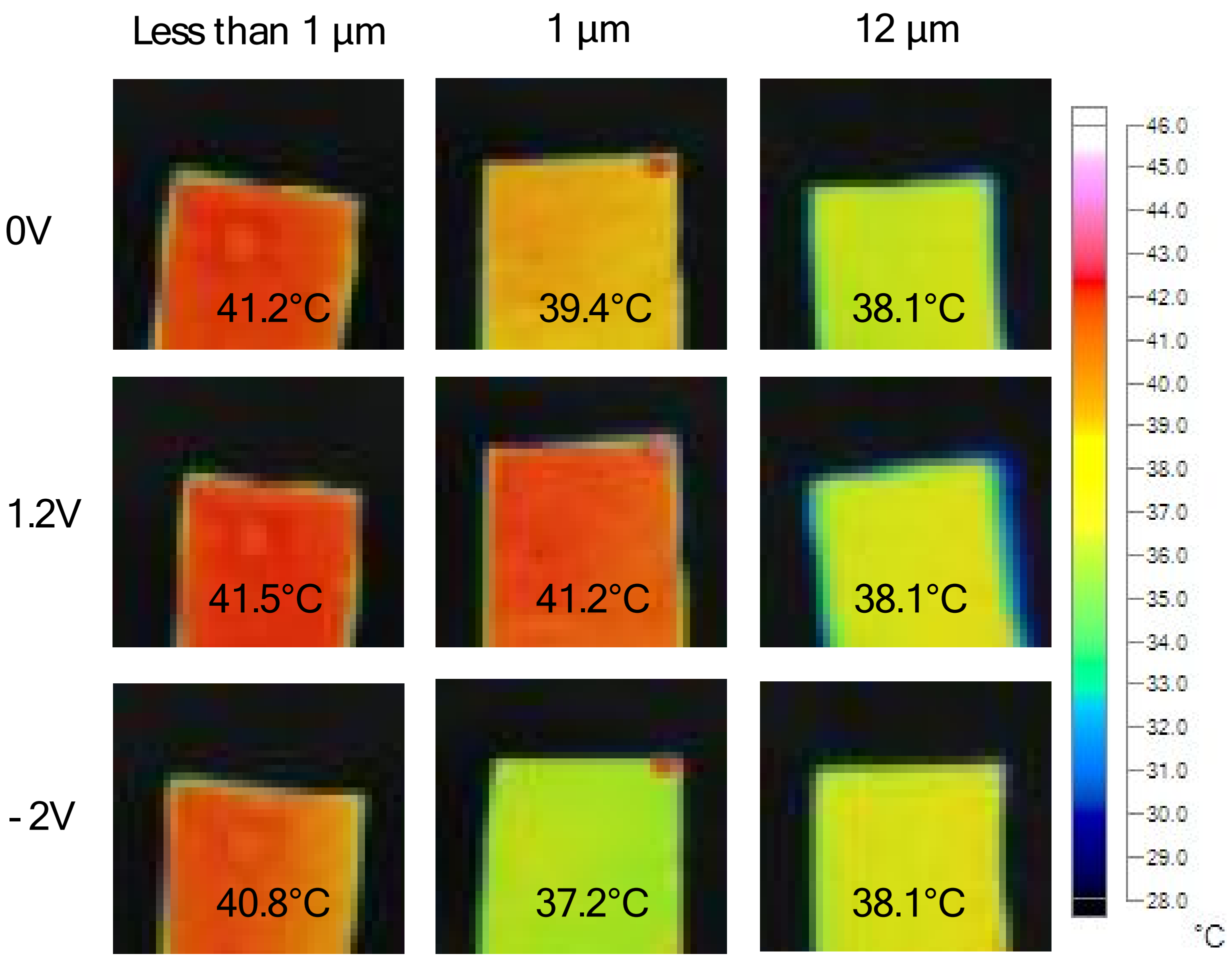

Figure S3. The dependence of tuning ability of the SWCNT emission modulator on the thickness of SWCNT film (body temperature at 42°C).

Table S1. The apparent temperature of the modulator surfaces with different SWCNT film thickness and gating voltages

Voltage \ Thickness	Less than 1μm	1μm	12μm
0	41.2°C	39.4°C	38.1°C
1.2V	41.5°C	41.2°C	38.1°C
-2V	40.8°C	37.2°C	38.1°C

Frequency response

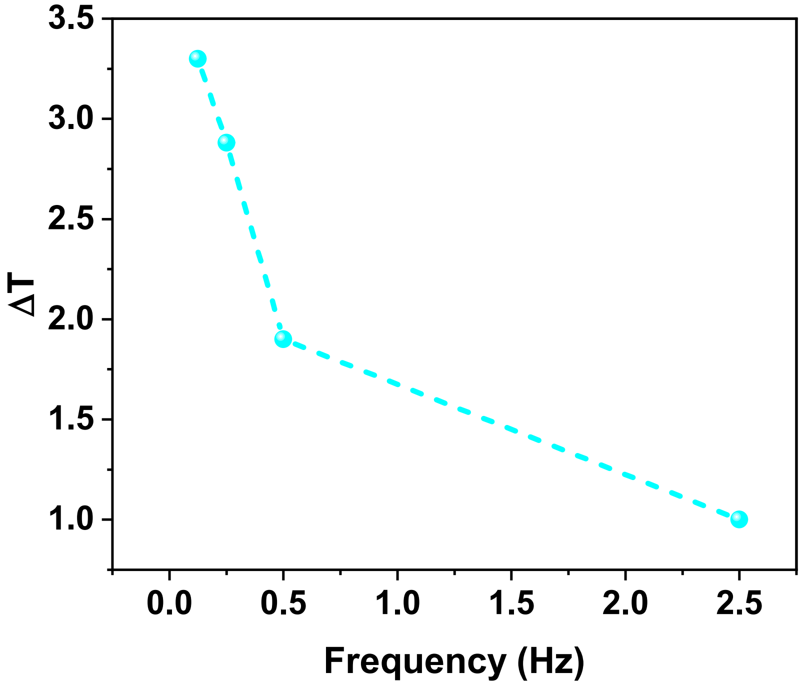

Figure S4. The dependence of tuning range with respect to the input tuning signal frequency.

References:

(1) Ariyoshi, S.; Hashimoto, S.; Ohnishi, S.; Negishi, S.; Mikami, H.; Hayashi, K.; Tanaka, S.; Hiroshiba, N. Broadband Terahertz Spectroscopy of Cellulose Nanofiber-Reinforced Polypropylenes. *Mater. Sci. Eng. B Solid-State Mater. Adv. Technol.* **2021**, *265*, 115000. https://doi.org/10.1016/j.mseb.2020.115000.

(2) Kaland, H.; Håskjold Fagerli, F.; Hadler-Jacobsen, J.; Zhao-Karger, Z.; Fichtner, M.; Wiik, K.; Wagner, N. P. Performance Study of MXene/Carbon Nanotube Composites for Current Collector- and Binder-Free Mg–S Batteries. *ChemSusChem* **2021**, *14* (8), 1864–1873. https://doi.org/10.1002/cssc.202100173.
